# Novel circulating tumor cell-detection chip combining conventional podoplanin and EGFR antibodies for all histological malignant pleural mesothelioma

**DOI:** 10.3892/ol.2021.12783

**Published:** 2021-05-11

**Authors:** Masatoshi Kanayama, Rintaro Oyama, Masataka Mori, Akihiro Taira, Shinji Shinohara, Taiji Kuwata, Masaru Takenaka, Kazue Yoneda, Koji Kuroda, Takashi Ohnaga, Yukinari Kato, Fumihiro Tanaka

**Affiliations:** 1 Second Department of Surgery, University of Occupational and Environmental Health, Kitakyushu, Fukuoka 807-8555, Japan; 2 Central Research Laboratories, Toyama Industrial Technology Center, Takaoka, Toyama 933-0981, Japan; 3 Department of Antibody Drug Development, Tohoku University Graduate School of Medicine, Sendai, Miyagi 980-8575, Japan; 4 New Industry Creation Hatchery Center, Tohoku University, Sendai, Miyagi 980-8575, Japan

**Keywords:** circulating tumor cells, malignant pleural mesothelioma, CTC-chip, podoplanin, epidermal growth factor receptor

## Abstract

In our previous study, a microfluidic system was developed based on podoplanin detection for capturing circulating tumor cells (CTCs), derived from malignant pleural mesothelioma (MPM). However, non-epithelioid MPM shows low podoplanin protein expression compared with that in epithelioid MPM; thus, some CTC populations may be missed. To overcome this limitation, a new CTC-detection chip was developed by combining the conventional podoplanin antibody (clone: NZ-1.2) with an epidermal growth factor receptor (EGFR)-targeted antibody (cetuximab). The cell-capture efficiency of the Cocktail-chip reached 100% in all the histological MPM cell lines. The median CTC-counts from 19 patients with MPM (epithelioid/non-epithelioid: 10/9) with the NZ-1.2- and Cocktail-chips were 1 and 3 (P=0.311) in 1 ml peripheral blood, 1.5 and 2 (P=0.332) in epithelioid MPM, and 1 and 3 (P=0.106) in non-epithelioid MPM, respectively. Overall, the Cocktail-chip showed an improved ability to detect more CTCs in patients with non-epithelioid MPM compared with that in the conventional NZ-1.2-chip, showing non-significant, but higher CTC detection. Furthermore, CTC-counts, determined using the Cocktail-chip were significantly correlated with the clinical stage of non-epithelioid MPM. In epithelioid MPM, the Cocktail-chip achieved a CTC-detection efficiency equivalent to that in the conventional NZ-1.2-chip. The Cocktail-chip enabled sensitive CTC detection of all histological MPM, including the non-epithelioid subtype, which may provide a foundation for the diagnosis, treatment, and prognosis of MPM progression.

## Introduction

Circulating tumor cells (CTCs) are derived from the primary tumor and circulate in the peripheral blood (1). They are considered to play a crucial role in metastasis formation, which is the leading cause of cancer-related death. Therefore, the detection and molecular biological analysis of CTCs may enhance the diagnosis and treatment of patients with cancer (2–4). Among the various CTC-detection devices currently available, only the CellSearch system (Menarini Silicon Biosystems Spa) has been approved by the Food and Drug Administration for clinical use (5), and has produced highly reproducible results and demonstrated clinical relevance in breast, colorectal, prostate and lung cancer (6–11). However, as CellSearch is an epithelial cell adhesion molecule (EpCAM)-dependent cell-capture system, it fails to identify CTCs in non-epithelial tumors with low EpCAM expression, such as malignant pleural mesothelioma (MPM) (12). To overcome this diagnostic gap, in our previous studies, a new microfluidic device system, named as the ‘Universal CTC-Chip’, was developed, which enables EpCAM-independent cell capture by attaching various antibodies to a large number of microposts on the chip surface (13,14). In addition, the clinical significance of CTCs in MPM was found and CTC capture was possible using an antibody against podoplanin, a well-known diagnostic marker of MPM (14–16). However, podoplanin expression is lower in non-epithelioid MPM (30-75%) compared with that in epithelioid MPM (80-100%) (17–20), which may reduce the efficiency of this diagnostic approach. Furthermore, our previous study showed that CTCs were detected in 92.3% (12/13 patients) of epithelioid MPM cases compared with that in 33.3% (3/9 patients) of non-epithelioid MPM cases (16), suggesting that some CTC populations do not express podoplanin, particularly those of the non-epithelioid subtype. Epidermal growth factor receptor (EGFR) is a 170 kDa transmembrane protein with intrinsic tyrosine kinase activity that regulates cell growth (21). EGFR is overexpressed in several malignancies, including non-epithelioid MPM (22–25). In recent years, cetuximab, a chimeric antibody targeting EGFR, was found to be an effective cell-capture antibody compared with that in other EGFR antibodies, due to its low dissociation constant and strong cell adhesion ability (26). In the present study, the following was investigated: i) EGFR expression in the MPM cell lines; ii) the cell-capture efficiency of a CTC-chip coated with cetuximab; and iii) whether an antibody cocktail of podoplanin (clone NZ-1.2) and cetuximab could enhance CTC capture in all histological subtypes of MPM.

## Materials and methods

This research was conducted with the approval of the Ethics Committee of the University of Occupational and Environmental Health, Japan (approval no. H26-15).

### 

#### Cell lines

A total of 5 human cell lines representing different histological subtypes of MPM were used: Epithelioid MPM: ACC-MESO-1, ACC-MESO-4, and NCI-H226; biphasic MPM: MSTO-211H; and sarcomatoid MPM: NCI-H28. The ACC-MESO-1 and ACC-MESO-4 cell lines were purchased from the Riken BioResource Research Center, while the NCI-H226, MSTO-211H, and NCI-H28 cell lines were purchased from the American Type Culture Collection. All the cell lines were cultured in RPMI-1640 (FUJIFILM Wako Pure Chemical Corporation), supplemented with 10% fetal bovine serum (Thermo Fisher Scientific, Inc.) at 37°C in a humidified incubator with 5% CO_2_.

#### Flow cytometry analysis

To analyze EGFR expression, the cell lines were incubated with an anti-EGFR antibody (1:100 dilution; clone 528; cat. no. sc-120; Santa Cruz Biotechnology, Inc.) for 1 h at room temperature. The cells were subsequently incubated with goat anti-mouse IgG antibody conjugated with fluorescein isothiocyanate for 30 min at room temperature (1:20 dilution; cat. no. 349031; BD Biosciences). Flow cytometry analysis was performed using an EC800 Cell Analyzer (Sony Biotechnology, Inc.), and the data was analyzed using FlowJo software version 10 (Becton-Dickinson and Company). The mean fluorescence intensity was calculated as the ratio of the positive control to that of the negative control (PBS with 1% bovine serum albumin; Nacalai Tesque, Inc.).

#### CTC-chip preparation

The CTC-chip was first coated with antibodies in a two-step process as previously described (14), then used for the experiments. Briefly, the CTC-chip was incubated with the base antibody overnight at 4°C followed by incubation with the capture antibody at room temperature for 1 h.

In the present study, the CTC-chip was built based on the following three combinations: i) previously established NZ-1.2-chip (16) with goat anti-rat IgG (200 µg/ml; cat. no. 3052-01; SouthernBiotech) as the base antibody and rat anti-human podoplanin antibody (5,000 µg/ml; clone NZ-1.2) (27) as the capture antibody; i) Cetuximab-chip with goat anti-human IgG (200 µg/ml; cat. no. 2043-01; SouthernBiotech) as the base antibody and cetuximab (5,000 µg/ml; Bristol-Myers Squib Company) as the capture antibody and iii) Cocktail-chip with goat anti-rat IgG (200 µg/ml) + goat anti-human IgG (200 µg/ml) as base antibodies and rat anti-human podoplanin antibody (5,000 µg/ml) + cetuximab (2,500 µg/ml) as capture antibodies.

#### Sample preparation and evaluation of cell-capture efficiency in the MPM cell lines

Sample preparation and evaluation of cell-capture efficiency was performed as previously described (14). Tumor cells labeled using the CellTrace CSFE cell proliferation kit (Thermo Fisher Scientific, Inc.) were suspended in 1 ml blood collected from a healthy volunteer (obtained from author MK; single venous blood collection from the elbow; 100 cells/ml). This sample was added to each CTC-chip system at a constant flow rate (1.0 ml/h) and monitored using a fluorescence microscope (CKX41; Olympus Corporation). The total number of cells added to the CTC-chip (N-total) was determined by counting the number of cells that passed through the inlet of the CTC-chip, whereas the number of captured cells (N-captured) was determined by counting the number of cells that remained on the CTC-chip. Cell-capture efficiency was calculated as the N-captured/N-total ×100 (%), and the mean ± SE was calculated for each sample. Each experiment was performed in triplicate.

#### Clinical evaluation of CTCs in patients with MPM

Peripheral blood samples were collected from 19 patients with MPM between January 2018 and August 2020, to assess CTCs at diagnosis or prior to treatment. A total of 6 ml blood was collected in a collection tube (BD Vacutainer EDTA-2K; BD Biosciences), and after sufficient suspension, 1 ml blood was added to both the NZ-1.2- and Cocktail-chips. The characteristics of the patients with MPM are summarized in Table I. Clinical stage was determined according to the guidelines of the International Mesothelioma Interest Group, version 8 (28). All patients provided written informed consent to participate in the study. The cells captured on 2 of the CTC-chips were incubated with a primary antibody, a rabbit anti-cytokeratin (CK) antibody (1:100 dilution; cat. no. ab9377; Abcam) and a mouse anti-CD45 antibody (1:100 dilution; cat. no. 304002; clone HI30; BioLegend, Inc.) for 1 h at room temperature, followed by incubation with 30 min of incubation at room temperature with a secondary antibody, an Alexa Fluor 594 anti-rabbit IgG antibody (1:100 dilution; cat. no. A-11037; Thermo Fisher Scientific, Inc.) and an Alexa Fluor 488 anti-mouse IgG antibody (1:100 dilution; cat. no. A-11029; Thermo Fisher Scientific, Inc.) containing 1 µg/ml Hoechst 33342 (cat. no. 4082; Cell Signaling Technology, Inc.) Cells with round-to-oval morphology, Hoechst 33342-positive nuclei, CK-positive staining in the cytoplasm, and CD45-negative staining were identified as CTCs using a fluorescence microscope at ×10 magnification (DMi8-S2G; Leica Microsystems), and CTCs were independently identified by two investigators who were blinded to the clinical data. Survival analysis according to the CTC-counts in NZ-1.2-chip and Cocktail-chip was also performed. The median follow-up was 175 (range, 28–1,067) days.

In addition, to evaluate non-specific detection, peripheral blood samples were collected from five healthy individuals (6 ml; single venous blood collection from the elbow) and used to detect CTCs in the same manner as aforementioned. The healthy volunteers were recruited from our laboratory staff and provided formal written informed consent to participate. Data collection from the healthy subjects was also included in the Ethics Committee approval (approval no. H26-15).

#### Evaluation of podoplanin and EGFR expression using immunohistochemical staining in the primary lesions

Serial 4-µm-thick sections were cut from each 10% formalin-fixed and paraffin-embedded primary tumor specimen collected by pleural biopsy or radical surgery, then evaluated using immunohistochemistry staining according to standard protocols. Sections were heated in 0.01 M citrate buffer (pH 6.0; cat. no. RM102-C; LSI Medience Corporation) at 98°C for 15 min for antigen retrieval and incubated in 3% hydrogen peroxide (cat. no. 081-04215; FUJIFILM Wako Pure Chemical Corporation) for 10 min to inactivate endogenous peroxidase. After blocking with Protein Block Serum-Free (cat. no. X090930-2; Agilent Technologies, Inc.) for 15 min, sections were incubated with mouse anti-podoplanin monoclonal antibody (clone D2-40; cat. no. 413451; pre-diluted antibody; Nichirei Biosciences, Inc.) and rabbit anti-EGFR monoclonal antibody (1:50 dilution; clone D38B1; cat. no. 4267S; Cell Signaling Technology, Inc.) for 1 h. Sections were then washed and incubated with Histofine Simple Stain MAX PO (MULTI) (cat. no. 424152; Nichirei Biosciences, Inc.) for 30 min. Thereafter, sections were visualized with DAB+ Liquid (cat. no. K346811; Agilent Technologies, Inc.) for 10 min and counterstained with hematoxylin for 1 min (cat. no. 30002; Muto Pure Chemicals Co., Ltd.). All steps after the antigen retrieval step were performed at room temperature.

#### Statistical analysis

Differences between continuous variables were evaluated using a Wilcoxon signed rank test for paired data, that was not normally distributed. The correlation between two variables was analyzed using Spearman's rank correlation analysis. The Kaplan-Meier method was used to estimate the probability of survival, with survival differences being analyzed using the log-rank test. P<0.05 was considered to indicate a statistically significant difference. All statistical analyses were performed using SPSS software (version 27.0; IBM Corp.).

## Results

### 

#### Cell-capture efficiency of the three types of CTC-chip

**Figure 1.**
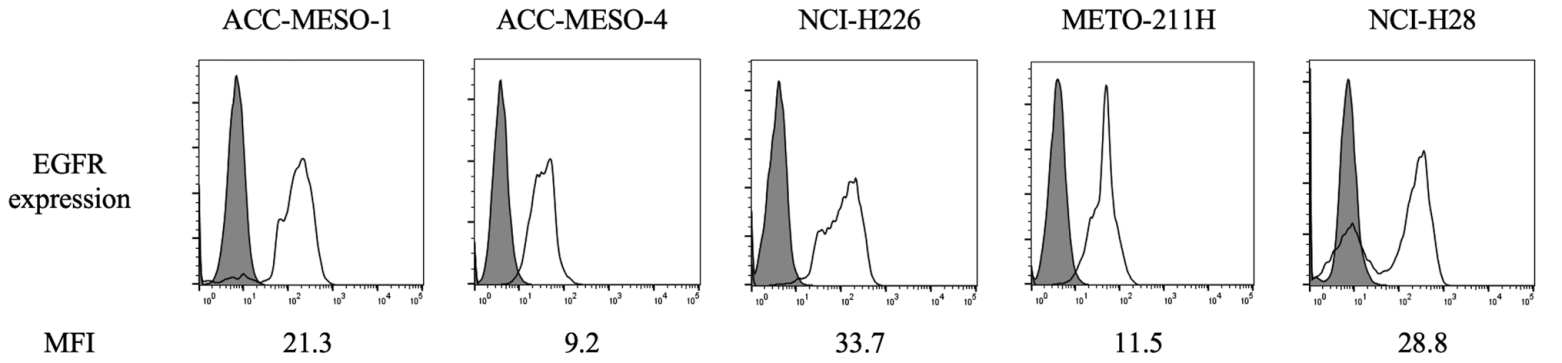
Expression of EGFR and podoplanin on the surface of malignant pleural mesothelioma cell lines detected using flow cytometry. EGFR, epidermal growth factor receptor; MFI, mean fluorescence intensity.

The flow cytometry results of EGFR expression are shown in Fig. 1. EGFR was detected in all the cell lines, including biphasic and sarcomatoid MPM subtypes, which have low podoplanin expression.

**Figure 2.**
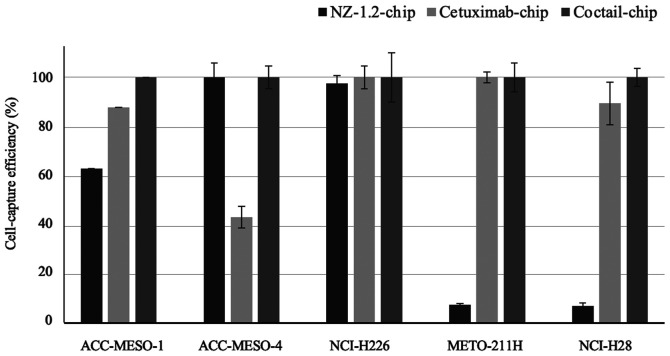
Cell-capture efficiency of the NZ-1.2-, cetuximab- and Cocktail-chips in malignant pleural mesothelioma cell lines.

The optimal concentration of cetuximab to be used on the newly designed CTC-chip was determined using the NCI-H226 cell line. The cell-capture efficiencies were 89.2, 89.1 and 102.5% at cetuximab concentrations of 500, 1,000 and 5,000 µg/ml, respectively (data not shown). Therefore, 5,000 µg/ml was used as the optimal cetuximab concentration for the further experiments. The cell-capture efficiencies of the three types of CTC-chips are shown in Fig. 2. The epithelioid MPM cell lines, with high podoplanin expression, were effectively captured by the NZ-1,2-chip containing podoplanin, but the cell-capture efficiency for the non-epithelioid cell lines was low. The Cetuximab-chip, containing the anti-EGFR antibody, had high cell-capture efficiency in the majority of the cell lines, except for the ACC-MESO-4 cell line, in which there was a 40% cell-capture efficiency. The Cocktail-chip, containing antibodies targeting both podoplanin and cetuximab, reached 100% of a cell-capture efficiency in all the MPM cell lines; therefore, it was used for clinical evaluation.

#### Clinical evaluation of the Cocktail-chip in patients with MPM

**Figure 3.**
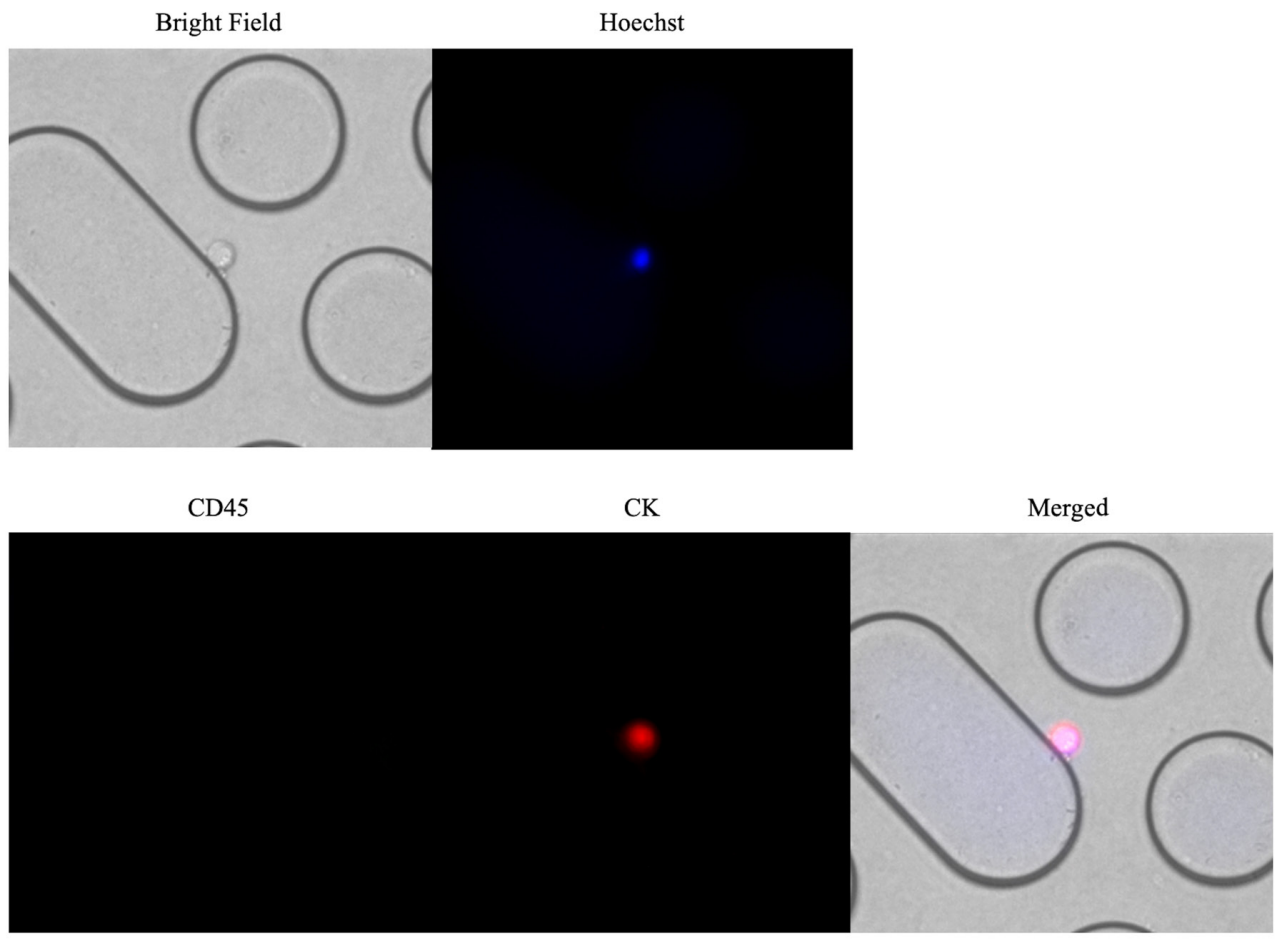
Representative images of immunofluorescent staining of CTCs captured using the Cocktail-chip in patients with malignant pleural mesothelioma. CTCs, circulating tumor cells.

**Figure 4.**
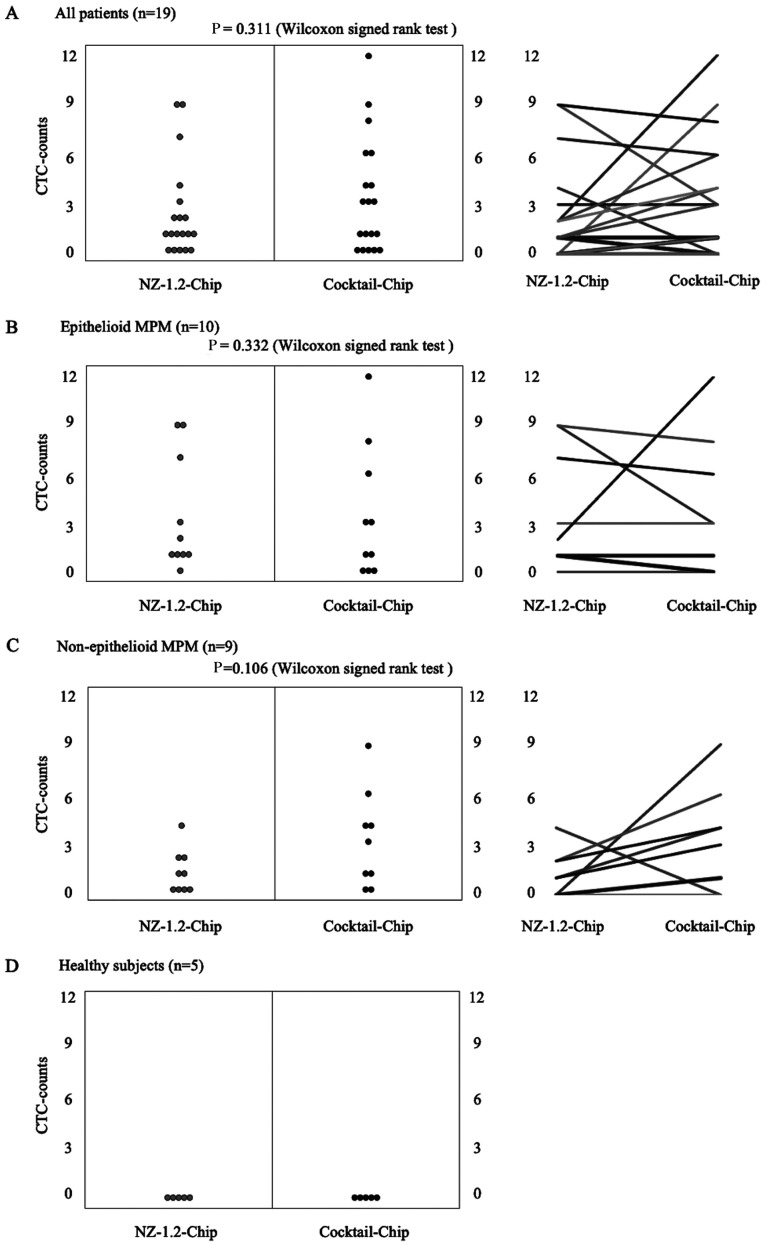
Wilcoxon signed rank analysis of CTCs detected using the NZ-1.2- and Cocktail-chips according to their count. The CTCs were analyzed from 1 ml peripheral blood collected from patients with (A) malignant pleural mesothelioma (all patients), (B) epithelioid, (C) non-epithelioid subtype and (D) healthy subjects. CTCs, circulating tumor cells.

Representative images of immunofluorescent staining of CTCs captured using the Cocktail-chip in patients with malignant pleural mesothelioma are shown in Fig. 3. Fig. 4 shows the distribution of CTC-counts using the NZ-1.2- and Cocktail-chips for each sample. No cells were detected in the five healthy subjects, and no non-specific detection was observed (Fig. 4D). CTCs were detected in 73.7% of the samples (14/19 patients) with both the NZ-1.2- and Cocktail-chips. Furthermore, the NZ-1.2- and Cocktail-chips detected CTCs in 90 (9/10 patients) and 70% (7/10 patients) of samples with epithelioid MPM, and in 55.6 (5/9 patients) and 77.7% (7/9 patients) of samples with non-epithelioid MPM, respectively. No clusters, such as clusters of tumor cells or clusters of tumor cells and white blood cells, were observed. The median CTC-counts using the NZ-1.2- and Cocktail-chips were 1 (range, 0–9) and 3 (range, 0–12) in the overall population (P=0.311), 1.5 (range, 0–9) and 2 (range, 0–12) in epithelioid MPM (P=0.332), and 1 (range, 0–4) and 3 (range, 0–9) in non-epithelioid MPM (P=0.106), respectively. There was no significant difference between the two chips; however, the Cocktail-chip detected more CTCs in non-epithelioid MPM, suggesting it was more effective at detecting CTCs. In addition, the Cocktail-chip achieved a CTC-detection efficiency equivalent to that of the conventional NZ-1.2-chip in epithelioid MPM, that is there was no change in the number of epithelial cells (Fig. 4B).

**Figure 5.**
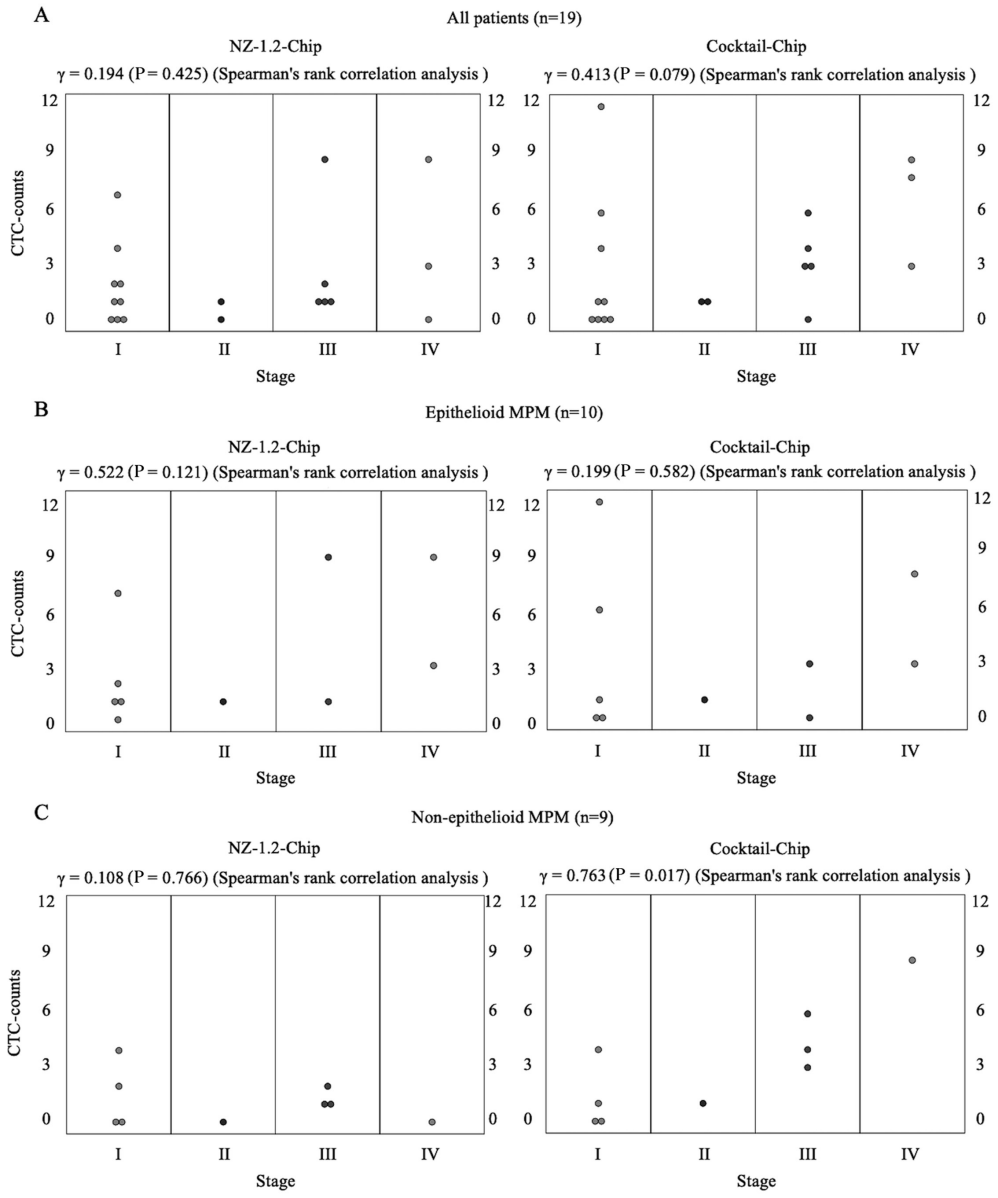
Spearman's correlation analysis of CTCs detected using the NZ-1.2- and Cocktail-chips according to their count and clinical stage. The CTCs were analyzed from 1 ml peripheral blood collected from patients with (A) malignant pleural mesothelioma (all patients), (B) epithelioid, and (C) non-epithelioid subtype. CTCs, circulating tumor cells.

The correlation coefficient between CTC-counts and clinical stage in all patients was 0.194 (P=0.425) with the NZ-1.2-Chip and 0.413 (P=0.079) with the Cocktail-chip. Based on the histology data, the correlation coefficients in epithelioid and non-epithelioid MPM were 0.522 (P=0.121) and 0.199 (P=0.582) for CTC-counts with the NZ-1.2-Chip and 0.108 (P=0.766) and 0.763 (P=0.017) with the Cocktail-chip, respectively (Fig. 5). These data indicated that the CTC-counts using the Cocktail-chip were significantly correlated with the clinical stage of non-epithelioid MPM.

**Figure 6.**
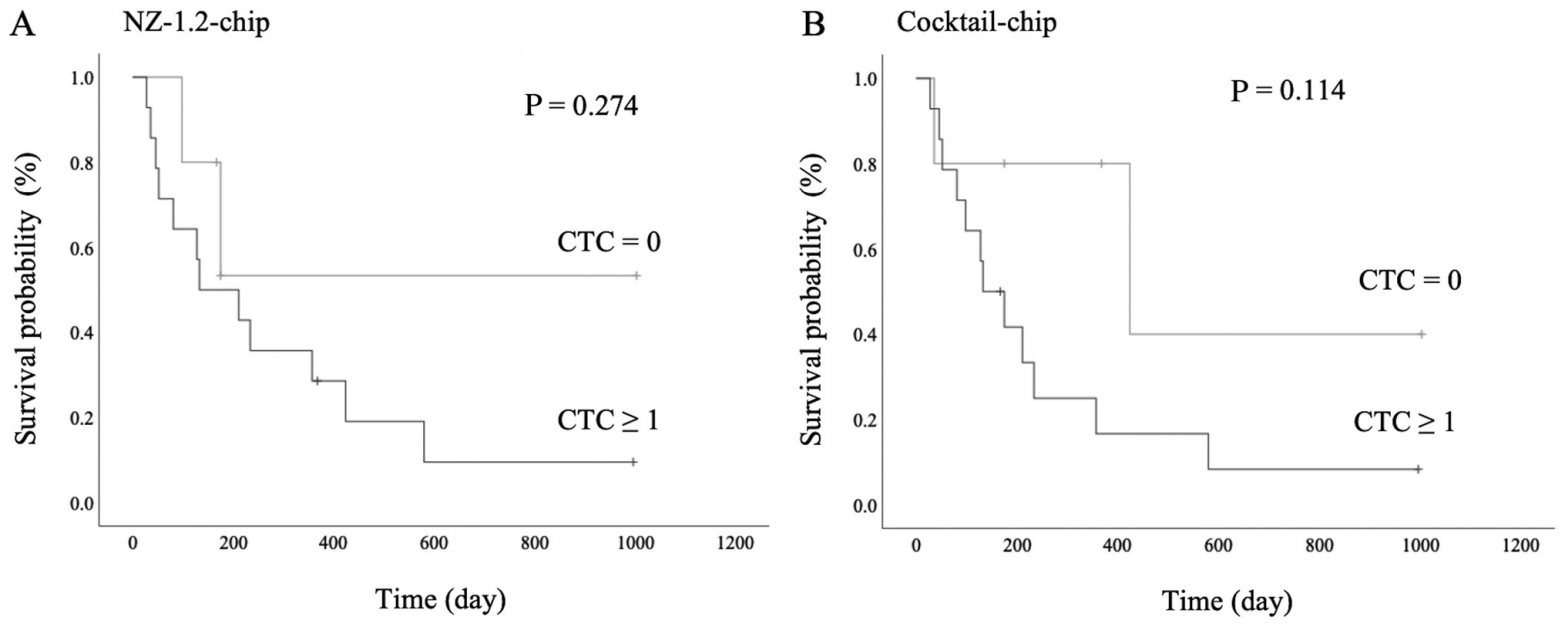
Survival analysis of CTCs in patients with malignant pleural mesothelioma. Kaplan-Meier survival curves and log-rank tests were used for the analysis in patients with no CTCs (0) and ≥1 CTCs using the (A) NZ-1.2- and (B) Cocktail-chips. CTCs, circulating tumor cells.

The survival analysis is shown in Fig. 6. There was no statistically significant difference between the presence of CTCs and prognosis in both the NZ-1.2- and Cocktail-chips; however, patients with CTCs detected had a poorer prognosis (P=0.274 and P=0.114, respectively). Furthermore, in patients with stage I MPM, one patient without CTCs detected survived without progression for >3 years following surgery, whereas 2 patients with CTCs detected showed early progression and died following treatment. More specifically, case 1 (NZ-1.2-chip:2, Cocktail-chip:12) died 4 months following chemotherapy due to tumor progression, including distant metastasis, and case 2 (NZ-1.2-chip:2, Cocktail chip:4) died 4 months following surgery due to locally advanced tumor progression (data not shown).

#### Evaluation of podoplanin and EGFR expression using immunohistochemical staining in primary lesions

**Figure 7.**
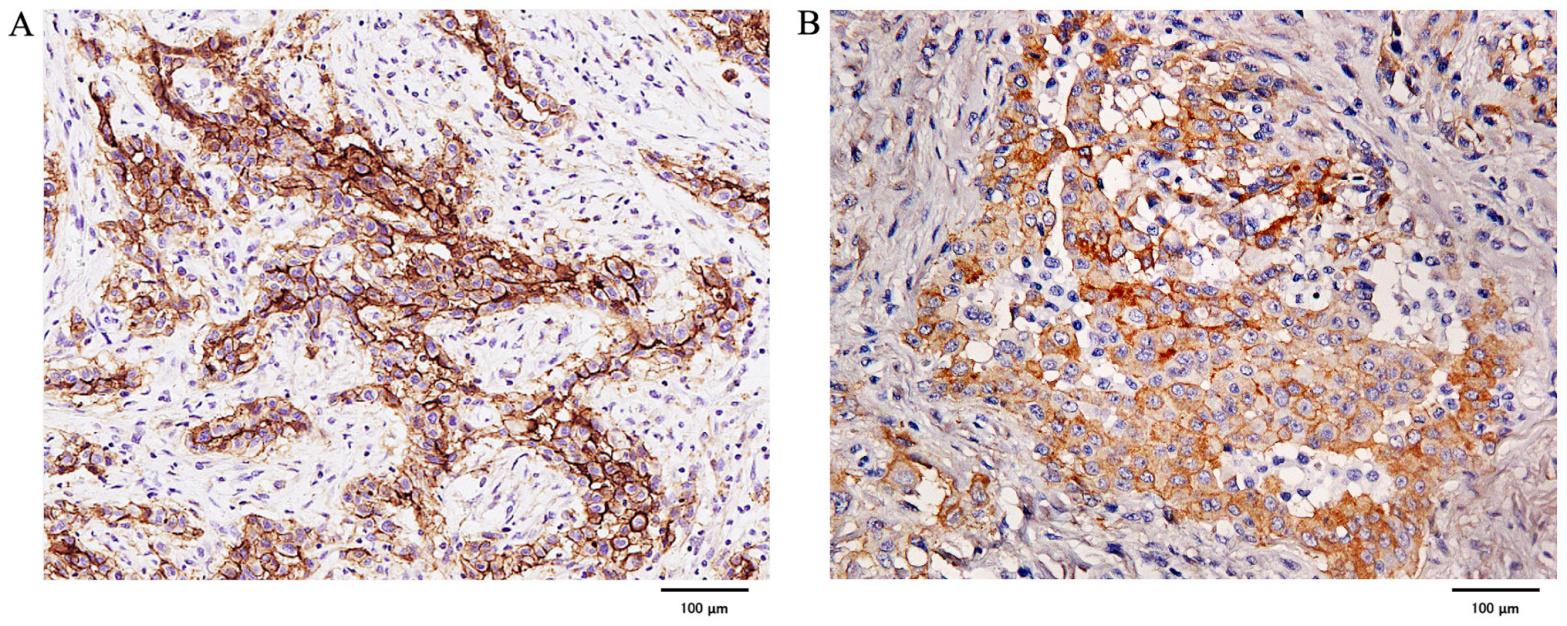
Immunohistochemical staining of the primary lesions in patients with malignant pleural mesothelioma. Staining of (A) podoplanin (clone D2-40) and (B) epidermal growth factor receptor (clone D38B1).

Podoplanin staining was strongly positive in all cases of epithelioid MPM, whereas it was negative or weakly positive in 3 out of 5 cases of sarcomatoid MPM. In biphasic MPM, the epithelial component was strongly positive in all four cases, whereas the sarcoma component was negative or weakly positive in some cases. In contrast, there were no negative cases of EGFR, and all were strongly positive except for 2 epithelioid and 1 biphasic MPM, which were weakly positive (Fig. 7).

## Discussion

In the present study, EGFR expression was confirmed in all types of the MPM cancer cell lines. By combining with cetuximab (Cocktail-chip), the cell-capture efficiency was higher in non-epithelioid MPM-derived CTCs compared with that in the conventional NZ-1.2-chip, which was designed to only detect podoplanin.

Effective capture of rare CTCs presents technical challenges. A common strategy is EpCAM-dependent isolation, as is used in CellSearch, because epithelial tumor cells express EpCAM. However, EpCAM-dependent methods may not be applicable to the isolation of non-epithelial tumor cells (15), and CTCs in MPM have not been fully investigated so far. In our previous studies, a CTC capture system for MPM using anti-podoplanin antibodies (clone E1) (14,15) and NZ-1.2 (16) was developed, and demonstrated its clinical significance. However, that system did not detect CTCs in non-epithelioid MPM, such as sarcomatoid and biphasic subtypes, which are particularly aggressive (29). Therefore, the present study focused on EGFR as it is widely expressed in several types of tumor, such as gastric, liver, colorectal and lung cancer (21). EGFR expression was observed in non-epithelioid MPM cell lines with poor podoplanin expression, indicating that EGFR represents a suitable target for CTC detection. The Cetuximab-chip showed high cell-capture efficiency; however, this chip exhibited a low capture efficiency for one epithelioid subtype cell line (ACC-MESO-4). Thus, to overcome this possible detection limitation of cetuximab, a new chip was designed by combining antibodies targeting podoplanin and cetuximab. This dual detection approach had a high capture efficiency in all histological MPM cell lines. In a clinical setting, the Cocktail-chip also achieved a high cell-capture efficiency, as well as correlation with clinical stage in non-epithelioid subtype MPM cases. In addition, the immunostaining results showed that there were several cases of poor expression of podoplanin in non-epithelioid MPM. These results suggested that cetuximab could be used in combination with podoplanin to support CTC detection in the non-epithelioid subtype. Furthermore, the Cocktail-chip achieved a CTC-detection efficiency equivalent to that of the conventional NZ-1.2-chip in epithelioid MPM. Therefore, the results from the present study suggested that the Cocktail-chip could be used for CTC detection in all histological MPM cases.

In the present study, the prognostic analysis was not sufficient, due to the low number of cases and the observation period was short in some cases. There was no association between CTC detection and prognosis; however, patients in which CTCs were detected there was a poorer prognosis, which may indicate that CTC detection could be of benefit in treatment selection, such as prioritizing chemotherapy over surgery in patients in which CTCs were detected in the early stage. In addition, distant metastasis is a rare progression event in MPM and mainly occurs due to local metastasis; however, based on the results for case 2 in the present study and a reported case in which an increased number of CTCs during CTC monitoring contributed to detection of local progression (16), CTC detection in MPM may indicate local progression as well as distant metastasis. These clinical findings should be verified in further studies with larger numbers of cases.

The present study has several limitations. First, only 19 patients were included in this study; therefore, the cohort is too small to draw a definitive conclusion on the efficiency of the Cocktail-chip. Second, CTC detection and counting was performed only once for each patient. Therefore, collecting peripheral blood samples from newly diagnosed and treated patients with MPM is currently ongoing, which will verify the prognosis and predictive value of the general CTC-detection system. Third, there may have been variability in the experiment as 1 ml blood from each patient was used for each chip, and the experimental protocol was not fully automated. Finally, in the current study, it was not validated whether the cells captured with the CTC-chip were true MPM cells. To resolve these system-related limitations, the application of a fully automated system using the automated pipetting system ‘EDR-24LX’ (BIOTEC Co., Ltd.) is being evaluated and developing a protocol for genetically analyzing cells captured by the CTC-chip using the micromanipulator ‘M-152’ (NARISHIGE Group).

In conclusion, the novel Cocktail-chip was more effective at capturing CTCs from all histological MPMs, including the non-epithelioid subtype. Further studies may reveal the clinical value of MPM-derived CTCs captured using the Cocktail-chip. Based on the results from the present study, this Cocktail-chip system may assist in the development of novel diagnostic, therapeutic and prognostic options for monitoring MPM progression.

## Figures and Tables

**Table I.** Patient characteristics (n=19).

Characteristic	Value
Mean age (range), years	69.0 (55–78)
Sex, n (%)	
Male	19 (100.0)
Female	0 (0.0)
Tumor laterality, n (%)	
Right	13 (68.4)
Left	6 (31.6)
TNM stage, n (%)	
IA	2 (10.5)
IB	7 (36.8)
II	2 (10.5)
IIIA	1 (5.3)
IIIB	4 (21.1)
IV	3 (15.8)
Histology, n (%)	
Epithelioid	10 (52.6)
Non-epithelioid	9 (47.4)
Biphasic	4 (44.4)
Sarcomatous	5 (55.6)

## Data Availability

The datasets used and/or analyzed during the current study are available from the corresponding author on reasonable request.

## Abbreviations

CTCs: circulating tumor cells
EGFR: epidermal growth factor receptor
EpCAM: epithelial cell adhesion molecule
MPM: malignant pleural mesothelioma
